# Cardiac diffusion tensor imaging approaching cellular length scales reveals striking microstructural detail

**DOI:** 10.1093/ehjimp/qyae043

**Published:** 2024-05-16

**Authors:** Irvin Teh, Erica Dall'Armellina, Jürgen E Schneider

**Affiliations:** Leeds Institute of Cardiovascular and Metabolic Medicine, University of Leeds, LIGHT Building, Clarendon Way, LS2 9NL, Leeds, UK; Leeds Institute of Cardiovascular and Metabolic Medicine, University of Leeds, LIGHT Building, Clarendon Way, LS2 9NL, Leeds, UK; Leeds Institute of Cardiovascular and Metabolic Medicine, University of Leeds, LIGHT Building, Clarendon Way, LS2 9NL, Leeds, UK

**Keywords:** diffusion tensor imaging, high-resolution, myocardial architecture, microstructure

Cardiac diffusion tensor imaging (DTI) is rapidly gaining traction in clinical research, but is constrained by limited resolution *in vivo*, commonly ∼2.3 × 2.3 × 8 mm^3^. To push boundaries, we imaged *ex vivo* mouse heart at 35 µm^3^ isotropic resolution, representing a million-fold smaller voxel size. Spin echo DTI was performed on a 7 T MRI scanner (scan time 76 h). For comparison, the data were also downsampled to 180 × 180 × 620 µm, to simulate clinical *in vivo* resolution scaled by a ∼13-fold size difference between human and mouse hearts, then interpolated to the original resolution. Primary eigenvector maps illustrate the dominant local cell orientations in whole heart (*Figure 1A*), with septal–lateral, anterior–posterior, and base–apex orientations in red, green, and blue, respectively. Features including crista terminalis and pectinate muscles are identified in high-resolution data (*Figure 1B*) but lost in lower resolution interpolated data (*Figure 1C*). A transmural helix angle gradient in the right ventricular wall mirroring the left ventricular wall (*Figure 1D*), aortic (*Figure 1E*), mitral, and tricuspid valves, and a complex arrangement of interdigitating cardiomyocyte bundles at the right ventricular insertion point (*Figure 1F*) are reported.

**Figure 1:**
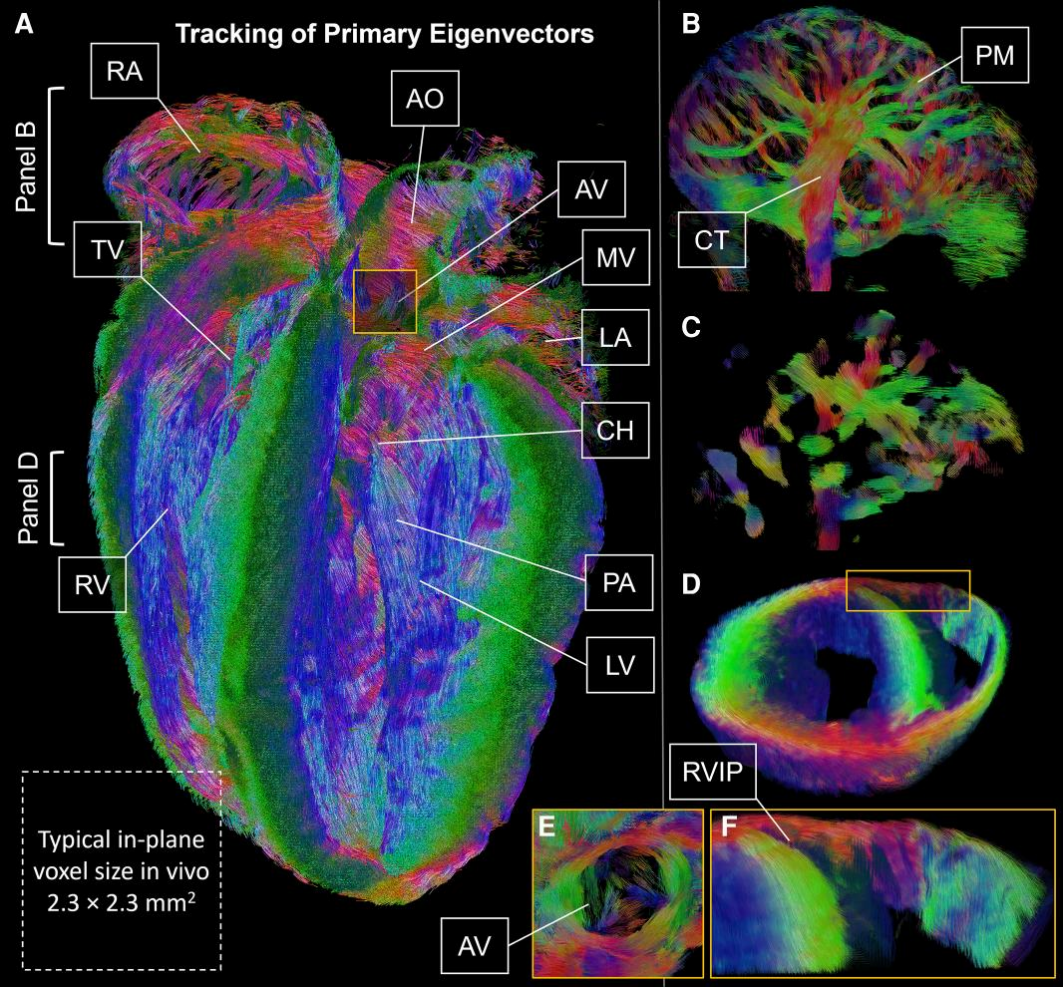


Ultra-high resolutions approaching cellular length scales can ameliorate partial volume, a major confound in DTI, particularly in finer structures with complex cell orientations. As demonstrated, DTI-informed cardiomyocyte orientations can yield substantially different results (related to induction, timing, and location of arrhythmias) in electrophysiological models compared with rule-based approaches. Thus, high-resolution murine data facilitate validation of biomechanical and electrophysiological simulations for subsequent translation to clinical application, paving the way for patient-specific modelling to improve health outcomes.

## Data Availability

The data underlying this article will be shared on reasonable request to the corresponding author.

## Lead author biography

Dr Irvin Teh is a senior research fellow and MRI lead for the Experimental and Preclinical Imaging Centre at the University of Leeds. Dr Teh is an MRI physicist who leads a group focused on developing advanced diffusion MRI methods for microstructural characterization of the heart. He is an investigator on grants from the British Heart Foundation, NIHR, and Wellcome Trust, and has published extensively on technical developments in advanced diffusion MRI, pre-clinical and clinical application of diffusion MRI, and validation of diffusion MRI using independent imaging techniques. Dr Teh currently co-chairs the SCMR cardiac diffusion special interest group.

